# Immunisation program reviews in East and Southern Africa: 2012-2018; key lessons

**DOI:** 10.11604/pamj.2020.37.385.27140

**Published:** 2020-12-30

**Authors:** Messeret Eshetu Shibeshi, Balcha Girma Masresha, Fussum Daniel

**Affiliations:** 1 World Health Organisation, Inter-Country Support Team for East and Southern Africa, Harare, Zimbabwe,; 2 World Health Organisation, Regional Office for Africa, Brazzaville, Congo

**Keywords:** Africa, immunisation, program review, EPI review, vaccine preventable diseases, data quality, hesitancy

## Abstract

**Introduction:**

World Health Organisation (WHO) recommends that countries conduct comprehensive national immunisation programme reviews regularly to help them identify systems wide-barriers or gaps and monitor performance against the set targets.

**Methods:**

we reviewed reports from the latest national immunisation program reviews conducted in the 20 countries in the subregion in the course of 2012-2018. We generated descriptive analysis of the findings across the subregion.

**Results:**

the 20 program reviews included field observations to the subnational levels as well as interviews with program staff and stakeholders. At the time of the reviews, only 11 countries had functional National Immunisation Technical Advisory Groups. Operational funding was inadequate in half of the countries. The reviews documented the cancellation of outreach services, supportive supervision visits and maintenance of cold chain equipment due to the lack of fuel or operational funding. Immunisation programs in 10 countries had major human resource gaps. Vaccine stock management tools were not effectively used in 10 countries, and stockout of vaccines and supplies was documented in 9 countries during the review. The full components of the Reaching Every District (RED) Strategy were implemented in only 3 of the 20 countries. Twelve countries reported challenges with the availability and accuracy of target populations. Four countries had documented the presence of vaccine hesitant groups at the time of the reviews.

**Conclusion:**

the reviews demonstrated challenges in various aspects of the programs in different countries. The implementation of the review recommendations should be built into the annual program plans, as well as into costed multi-year plans, in order to address the gaps and helps the program to attain the set targets. With the rapid evolution of the scope and complexity of the immunisation programs in recent years, countries should invest their efforts in building the capacity of their human resources as well as updating their logistics and data systems.

## Introduction

In May 2012, the World Health Assembly (WHA) endorsed the Global Vaccine Action Plan (GVAP) [1]. The World Health Organisation (WHO) African Region adopted a seven-year immunisation strategic plan (2014-2020) aligned to the GVAP [2]. Countries in the Region have developed and are implementing national strategic and annual action plans following the principles and targets of the GVAP and the Regional strategic plans. To determine progress towards the set milestones and targets, and to align program activities, countries regularly monitor vaccination coverage, disease trends and other programmatic indicators at national and subnational levels. As part of the effort to strengthen the governance and leadership of national immunisation programs, countries are expected to establish organs including the National Immunisation Technical Advisory Group (NITAG) comprised of independent technical experts responsible for generating evidence-based policy guidance and technical recommendations; the Inter-Agency Coordinating Committee (ICC) which brings the national authorities together with the various donors and technical partners, and is a platform for endorsing program plans, advocating and mobilizing resources; and the National Regulatory Authority (NRA) that ensures the appropriate registration of safe and quality vaccines for use in the country [3-5]. On the operational aspects, WHO advises countries to implement the Reaching Every District (RED) approach to be able to reach all communities and all children, including those who normally do not access health services, and thus to assure equity in immunisation service delivery. The RED approach comprises of establishing outreach services; conducting regular supportive supervision; linking services with communities; monitoring and using data for action; and the management of resources [6].

The monitoring of vaccination service delivery data is done by recording the number of doses of antigen delivered in each health facility and aggregating it at the district, provincial and national levels. Administrative coverage is calculated by dividing the number of doses provided by the target population (the number of surviving infants for the calendar year) for the particular catchment area. The target population is generated from census data or from projections made based on recent census data. Considering the challenge with the accuracy of numerator and denominator information, WHO and UNICEF generate annual estimates of coverage for each antigen for each member State using the provided administrative data, information from coverage surveys, logistics information and other programmatic data. These WHO estimates are only generated for the national level [7]. Data quality assessments are conducted regularly to assist countries to identify challenges with the data management system and be able to troubleshoot using the data quality [8]. WHO recommends the periodic conduct of comprehensive national immunisation programme reviews (EPI reviews) to help countries identify system-wide barriers or gaps, monitor performance against the set targets, and develop strategic direction to remove these barriers or gaps towards the development of a more robust and resilient program [9]. These program reviews, conducted every 4 to 5 years, constitute a holistic assessment of the strengths and weaknesses of the immunisation programme at the national, sub-national and service-delivery levels. The reviews are done with the participation of various technical partners, and provide the evidence to align the immunisation program´s strategic directions and priority activities which are then translated into comprehensive Multi Year Plans (cYMP), and subsequent annual program plans, and as advocacy tools to reinforce the engagement of the leadership within the respective Ministries of Health [8,9]. Countries supported with GAVI funding undertake annual joint appraisal exercises with a focus on grant monitoring and overall progress towards program goals. These appraisals do not substitute for comprehensive program reviews [10]. The Eastern and Southern African subregion of the WHO consists of 20 Member States, a subset of the 47-member State of the WHO African region. These countries are Botswana, Comoros, Eritrea, Eswatini, Ethiopia, Lesotho, Kenya, Madagascar, Malawi, Mauritius, Mozambique, Namibia, Rwanda, Seychelles, South Africa, South Sudan, Tanzania, Uganda, Zambia, and Zimbabwe. Between the years 2014 - 2018, all of the twenty countries in the sub-region conducted at least one comprehensive Immunisation Program review [11]. This manuscript attempts to summarise the strengths and weaknesses of the immunisation programmes in the countries in the subregion, as documented in the program reviews, with a view to highlight actions required for countries to progress towards the programmatic targets.

## Methods

Comprehensive Immunisation Program Reviews are cross-sectional program assessments that are conducted through the review of documents and data, the interview of immunisation program staff at national and subnational levels, as well as field visits to provinces, districts and to service delivery sites. The data from the program review is collected using standardised data collection tools developed by WHO AFRO, which are adapted to the individual country context. The tool includes sections that cover the following thematic areas: (i) Leadership, governance and coordination; (ii) Human resource management, capacity building and supervision; (iii) Vaccine supply quality, logistics and cold chain; (iv) Service delivery; (v) Data monitoring, immunisation quality and vaccine preventable diseases surveillance; (vi) Demand generation, social mobilisation and advocacy. At the end of a program review, the findings and the recommendations are compiled by the review team in a formal report and presented to the respective Ministry of Health. We reviewed the detailed narrative technical reports generated from each of the national immunisation program reviews conducted in the 20 countries in the subregion in the course of 2014-2018. We conducted a descriptive analysis of the findings from the review exercises and attempted to do a summary of the findings by thematic areas.

## Results

Each of the 20 countries conducted at one national immunisation program review between 2012 and 2018 (Table 1). These 20 program reviews included field observations to a total of 137 Regions, 314 districts and 718 health facilities. In addition, the reviews included interviews with 475 program staff and stakeholders from the national levels, 1,028 immunisation session observations, and also exit interviews with 1,435 care-givers. The field observations included verification of the existence of relevant plans, guidelines and tools, as well as supervision of immunisation services delivery, cold chain and vaccine management practices. The major findings from these reviews are summarised below.

**Table 1:** period of latest national comprehensive EPI program review by country; Eastern and Southern Africa subregion (2012-2018)

Country	Period of latest EPI review
Madagascar	Oct-12
Comoros	Oct-13
Lesotho	Jun-14
Zambia	Jul-14
Rwanda	Nov-14
Uganda	Mar-15
Tanzania	Jul-15
Malawi	Oct-15
Namibia	May-16
Eritrea	Aug-16
Mozambique	Aug-16
Zimbabwe	Aug-16
Eswatini	Nov-16
Seychelles	Apr-17
Botswana	Aug-17
South Sudan	Oct-17
South Africa	Nov-17
Kenya	May-18
Ethiopia	Jun-18
Mauritius	Jun-18

**Leadership, governance and coordination**: at the time of the program reviews, 16 of the countries (80%) had some sort of legislation promoting immunisation, and 13 (65%) had updated immunisation policy documents. All except one country had updated comprehensive multi-year plans for immunisation (cMYPs). Full government financing of the immunisation program was documented in the 6 countries while 14 countries (which were GAVI eligible) depended on complementary support from partners such as GAVI for new and underutilised vaccines and UNICEF for the purchase of traditional vaccines. Immunisation services were provided free of charge in public health institutions in all countries. In the area of coordination, only 11 countries had functional National Immunisation Technical Advisory Groups (NITAG), while 17 countries had National Regulatory Authorities (NRA) at the time of the review. The 6 self-financing countries did not have functioning Inter-Agency Coordination Committees (ICC), while in 10 of the remaining 14 countries, the ICC was playing a key role in the coordination of partner support to the national immunisation program. The level of coordination between the immunisation program and the unit responsible for disease surveillance was limited in 8 of the 20 countries. Operational funding was inadequate in half of the countries in the sub-region. The reviews documented the cancellation of outreach services as well as the regular conduct of supportive supervision and maintenance of cold chain capacity at sub national level in these countries, mainly related to the lack of fuel or funds to rent vehicles.

**Human resources management, capacity building and supportive supervision**: ten (50%) countries had gaps in the number of staff and the skill mix required to run an efficient immunisation program at national and sub-national levels. These countries also lacked clear terms of reference or job descriptions for the assigned focal persons who were engaged in the day-to-day management of the immunisation program at different levels. Seventeen countries (85%) had clear plans for program supervision. However, supervisory visits to the subnational levels were inadequate or irregular in 4 of these 17 countries, due to limitations in funding and means of transport. Standardised tools for the supervision of immunisation services were available in only 14 of the 20 countries.

**Vaccine and cold chain logistics**: vaccine stock management tools were not effectively used in 10 (50%) countries, with tools lacking or outdated or not used regularly to monitor vaccine stocks at the health facility levels. Stockout of vaccines and supplies at the sub national level, lasting more than 3 months, was documented in 9 (45.0%) countries during the review. It was documented that 4 countries had frequent power interruptions and inadequate backup generators. Five countries did not have an updated cold chain equipment inventory system and no cold chain rehabilitation/replacement plan at the time of the review. The cold chain capacity was considered inadequate according to the reviews in 4 countries mainly at the sub national level (Table 2).

**Table 2:** summary of findings on Vaccine Management and Cold Chain systems

Domain	Number	Percentage (%)
Reported stock out of vaccines and supplies of more than 3 months duration	9	45
Updated vaccine stock management tools and management system in place	10	50
Reliable power supply with adequate backup generators to support vaccine management at cold stores (at national level)	16	80
Updated cold chain equipment inventory, and rehabilitation /replacement plan	15	75
Adequate cold chain capacity	16	80

**Service delivery**: the full components of the RED Strategy were implemented in only 3 of the 20 countries (15%). At the time of the reviews, 14 countries (70%) did not have up-to-date operational level micro-plans for routine immunisation services. In addition, 9 (45.0%) countries were not able to organise outreach services as planned due to transport and field staff constraints. Five (25.0%) countries also did not have enough cold chain equipment to run outreach services. At the time of the reviews, three countries had not yet introduced AD syringes for use in their immunisation programmes. Defaulter tracing mechanisms were available in only 12 (60%) countries (Table 3).

**Table 3:** summary of findings on Immunisation Service Delivery

Domain	Number (n)	Percentage (%)
All components of RED strategy implemented	3	15
Interruption of outreach services due to shortage of transport, funding or human resource	11	55
Immunisation services not provided as scheduled; cancelations due to vaccine stock out, inadequate cold chain maintenance, etc.	5	25
AD syringes not yet in use in the immunisation system	2	10

**Coverage monitoring, disease surveillance and data quality**: the availability and accuracy of target populations has been reported as a major challenge in 12 (60.0%) out of the 20 countries. Besides, the review in ten countries detected gaps in the systematic use of data for decision making, especially at the district and health facility levels, linked mostly due to knowledge gaps and limited resources. Data quality review and data harmonisation meetings were held regularly in 13 (65%) countries. Seven countries reported major challenges with their monitoring systems. These included denominators projected from outdated census causing significant over or under-estimation of the real target population, too many monitoring tools, discrepancies in the data across different administrative levels, as well as limited time and capacity to analyse and use data for decision making (Table 4). The reviews in 10 of the countries also documented gaps among health workers in the basic knowledge required to run effective surveillance systems. These included gaps in the understanding of standard case definitions for priority conditions, the core performance indicators for the surveillance of Vaccine Preventable Disease (VPD) and the various epidemiological concepts in use in the surveillance system. All except one country had annual VPD surveillance plans at the time of the review. National expert committees for Adverse Events Following Immunisation (AEFI) were in place in 16 countries (Table 5).

**Table 4:** summary of findings on Immunisation Monitoring Systems

Domain	Number (n)	Percentage (%)
Reliable target population and denominator data not available at all levels for planning and monitoring	12	60%
Standardized and up-to-date monitoring tools available	16	80%
Data not used at service delivery point due to limited capacity of data analysis and use	10	50%
Data quality review not done to guide improvement; no data harmonization	7	35%
Limited or no tracking of defaulters at health facility level	8	40%
Updated monitoring charts being used for monitoring coverage	18	90%
Suboptimal quality of immunisation data (over and under reporting)	7	35%

**Table 5:** summary of findings on Vaccine Preventable Diseases Surveillance

Domain	Number (n)	Percentage (%)
Clear terms of reference available for subnational surveillance focal persons	15	75
Limitation of financial resources for VPD surveillance	8	40
Prioritization of surveillance sites being done regularly for active surveillance	15	75
Health workers knowledgeable on the standard reporting case definition of priority VPDs, surveillance monitoring indicators and outbreak definitions	10	50
Regular data review and harmonization not done between the immunisation, surveillance and lab teams	7	35
National surveillance guidelines available at health facility level	13	65
Inadequate monitoring of key surveillance quality performance indicators and disease trends	6	30
No surveillance work plan	1	5
Limited capacity of the National Public Health Laboratory	1	5

**Demand generation and social mobilisation**: fifteen countries had a communication strategy for the immunisation program. A written plan to address crisis /risk communication was available in 17 (85.0%) countries. In 5 countries, it was noted that communication efforts were well developed for new vaccines introduction and Supplemental Immunisation Activities (SIA) but quite weak in promoting routine immunisation and community surveillance. Four countries had documented the presence of vaccine hesitant groups at the time of the reviews. Four (20%) of the 20 countries did not have communication messages tailored to address specific groups.

## Discussion

The findings in this summary have been aggregated and no specific country detail is given, since the main objective of the study was to demonstrate the challenges countries face in general. Moreover, since the reviews took place in the years 2012-2018, it is expected that any gaps have been addressed by the specific countries following the review exercise. While 12 of the 20 reviews were conducted in the last 5 years, 40% were conducted more than 5 years ago indicating the need for timely program reviews to steer the programs in the right direction. Immunisation program reviews cover a broad scope of program area but may not always achieve the necessary depth given the limited time allocated to the exercise [8,9]. When in-depth reviews are needed, countries utilise the focused review tools that cover specific program areas which may include vaccine management practices, new vaccine introduction process and outcomes, surveillance performance, data quality or others [9,12]. The 20 program reviews covered in this study indicated that some countries did not have any legislation or policy framework for immunisation. Having the appropriate legislative framework helps countries to protect national immunisation budgets and also to address community demand for vaccines [13]. Similarly, the lack of functioning and vibrant inter-agency coordinating committees may affect the coordination of activities among the partners as well as the effective mobilisation of resources. Effective oversight and coordination of immunisation programmes by government and partners are critical to achieving national immunisation goals. National coordination forums, including ICCs and health sector coordinating committees play an essential role in this work [14,15].

The growing complexity of the immunisation area of work in the last two decades requires the presence of strong human resources with the necessary managerial skills and tools. Findings from the reviews show that half of the countries were experiencing limitations in their human resource capacity within the national program and at sub-national levels. Health worker training plays an important role in improving overall performance including vaccination coverage [16,17]. Other studies and program reviews have also shown the critical role of human resource in immunisation program delivery [18-20]. In the 20 program reviews in this sub-region, capacity gaps are also reflected in the lack of clear managerial tools (e.g., terms of reference, job descriptions and standard orientation/training for newly assigned focal persons). Inadequate supportive supervision and the absence of standard supervisory tools are other common challenges. Supportive supervision has been shown to be an important component in immunisation programmes [21,22]. Regarding vaccine management logistics, half of the countries in the sub-region were not using the standard stock management tools, which are critical for monitoring stock levels, vaccine utilisation, vaccine wastage, and forecasting needs. The reported stockouts of vaccine and supplies in nearly half of the countries in this review are important from the point of view of the missed opportunities for the respective antigens the gaps created. The finding is comparable to the study that documented 38% countries in sub-Saharan Africa experienced at least one national-level stockout event for at least one vaccine and for at least one month during 2015 [23]. In a study focused on the district level in 3 African countries, LaFond *et al*. have identified six common drivers of routine immunisation coverage improvement. These include the presence of a cadre of community-centered health workers, health system and community partnership, regular review of health workers performance, the delivery of immunisation services tailored to community needs, political commitment to routine immunisation, and the actions of development partners. The study claimed that the presence of all six factors together was found to have positive impact in terms of coverage improvements [24]. The finding from the 20 national immunisation program reviews indicated that not all of these enabling components of good quality immunisation program performance were being implemented at district level in some of the countries.

It is well known that service factors and parental attitudes and knowledge are some of the most important reasons for non-vaccination. Among the service factors, Favin *et al*. have found that geographic inaccessibility of services, vaccine stock-outs and/or cold chain problems accounted for the majority of missed opportunities for the vaccination of children in many countries [25]. A lot of challenges exist with regards to immunisation data monitoring and quality in the African region. This review has identified the main challenges including knowledge gaps, discrepancies in data at different levels, and weak capacity to use data for decision making. Some of the root causes of challenges in monitoring systems include the lack of sustainable resources for immunisation, logistical limitations, and the lack of reliable denominator data for planning and coverage monitoring [26]. The reviews documented serious gaps in the surveillance for Vaccine Preventable Diseases (VPDs) related to limited knowledge of health workers, inadequate resources and programmatic focus. Gaps were documented in the prioritisation for active surveillance, as well as in the monitoring and use of surveillance data, as reported in other studies in the African Region [27,28]. The presence of population groups hesitant towards vaccination documented in 4 countries is a growing challenge that has in the past contributed to disease outbreaks [29,30]. Noting the complexity of vaccine hesitancy and the limited evidence available on how it can be addressed, identified strategies should be carefully tailored according to the target population, their reasons for hesitancy and the specific context [31].

**Limitations of the study**: these national immunisation program reviews were conducted using similar approaches and tools, but the review was conducted by different teams and using tools adopted for the national context. This may result in differences in the formulation of the review questions and interpretation of data. During these reviews, the sampling of subnational units was done purposively to strike a balance between urban and rural districts as well as strong performing and weaker districts. Therefore, all the results may not be comparable across the countries. In addition, it is expected that the countries will have acted upon the review findings and made the necessary program changes since the reviews, and so the results may not reflect the current situation at country level at the time of publication.

## Conclusion

The national immunisation program reviews in the sub-region have been useful tools to identify barriers or program gaps and generate recommendations for the national program and local partners to address the barriers or gaps. The reviews in the subregion have demonstrated challenges with regards to various aspects of the programs in different countries and unless addressed they will fail short to ensure provision of equitable immunisation service delivery. The follow up of recommendations to address the challenges and gaps constitutes an essential component of the review exercise aiming to enable them to attain the set targets. Countries should invest in continuous and systematic capacity building, in the context of the continuously evolving and expanding immunisation program. In this context, we also recommend that countries should plan for and conduct EPI program reviews at least every 4-5 years, preferably timed to provide inputs into program plans along with setting up mechanisms to regularly monitor program performance and address gaps, as well as to follow up and implement recommendations from review exercises. These EPI reviews may not constitute an in-depth evaluation of all program components, indicating the need to tailor the implementation of the reviews towards specific aspects of the program, based on the country context and preliminary findings. In addition, as necessary, countries may plan to conduct in-depth evaluations and root cause analyses.

### What is known about this topic

- Comprehensive national immunisation program reviews are conducted once every 4 to 5 years in low- and middle-income countries, in order to identify program areas for improvement, realign program focus as necessary and prepare the program for the introduction of additional program elements;
- Comprehensive program reviews are complementary to reviews with in-depth focus on specific program components;
- Recommendations generated by program reviews help countries to focus their efforts to address major program gaps.

### What this study adds

- With the rapid evolution of the scope and complexity of the immunisation programs across the Region, countries should build in methods to regularly review program performance and develop their strategic directions with the required investments for program sustainability and also invest in capacity building;
- A significant proportion of national immunisation programs in the subregion continue to have gaps in their implementation of the Reaching Every District Approach, in their human resource capacity, as well as in their logistics and data management systems;
- The follow up of the review recommendations will be critical to address the identified system-wide barriers or gaps in order to strengthen the immunisation program and enable it to attain the objectives.
